# Ononin ameliorates inflammation and cartilage degradation in rat chondrocytes with IL-1β-induced osteoarthritis by downregulating the MAPK and NF-κB pathways

**DOI:** 10.1186/s12906-022-03504-5

**Published:** 2022-01-27

**Authors:** Fang Xu, Liang-Jun Zhao, Ting Liao, Zhao-Cong Li, Lei-Lei Wang, Pan-Yu Lin, Rui Jiang, Qing-Jun Wei

**Affiliations:** 1 Department of Orthopaedics Trauma and Hand Surgery, The First Affiliated Hospital of Guangxi Medical University, Shuangyong Road No. 6, Nanning, 530021 China; 2 Guangxi Colleges and Universities Key Laboratory of Prevention and Control of Highly Prevalent Diseases, Guangxi Medical University, Shuangyong Road No. 22, Nanning, 530021 China; 3 Department of Bone and Joint Surgery, The First Affiliated Hospital of Guangxi Medical University, Nanning, 530021 China; 4 Department of Endocrinology, Liuzhou Municipal Liutie Central Hospital, Feie Road No. 22, Liuzhou, 545007 China; 5 Department of Toxicology, School of Public Health, Guangxi Medical University, Shuangyong Road No. 22, Nanning, 530021 Guangxi China

**Keywords:** Ononin, Osteoarthritis, Chondrocyte, Inflammation, MMP-13, Collagen II, MAPK, NF-κB

## Abstract

**Background:**

Osteoarthritis (OA) treatment aims to improve inflammation and delay cartilage degeneration. However, there is no effective strategy presently available. Ononin, a representative isoflavone glycoside component extracted from natural Chinese herbs, exerts anti-inflammatory and proliferative effects. However, the therapeutic effect of ononin on chondrocyte inflammation remains unclear.

**Methods:**

In this study, we explored the therapeutic effect and potential mechanism of ononin in OA by establishing an interleukin-1 beta (IL-1β)-induced chondrocyte inflammation model.

**Results:**

Our results verified that ononin alleviated the IL-1β-induced decrease in chondrocyte viability, attenuated the overexpression of the inflammatory factors tumour necrosis factor α (TNF-α) and interleukin 6 (IL-6), and simultaneously inhibited the expression of cartilage extracellular matrix (ECM)-degrading enzymes such as matrix metalloproteinase-13 (MMP-13). Furthermore, the decomposition of Collagen II protein could be alleviated in the OA model by ononin. Finally, ononin improved chondrocyte inflammation by downregulating the mitogen-activated protein kinase (MAPK) and nuclear factor kappa-B (NF-κB) signalling pathways.

**Conclusion:**

Our findings suggested that ononin could inhibit the IL-1β-induced proinflammatory response and ECM degradation in chondrocytes by interfering with the abnormal activation of the MAPK and NF-κB pathways, indicating its protective effect against OA.

**Supplementary Information:**

The online version contains supplementary material available at 10.1186/s12906-022-03504-5.

## Introduction

OA is a chronic degenerative joint disease characterized by ECM degeneration, subchondral bone sclerosis, syndesmophyte formation, and exacerbated joint destruction in the late stage of disease, resulting the loss of the patient’s ability to work [1]. Although ageing and wear have been considered the main causes of this degenerative disease, current research provides a new view of cartilage degeneration caused by low-grade inflammatory responses [2, 3]. Proinflammatory cytokines and chemokines are produced in OA patient cartilage, subchondral bone and synovium, leading to the release of matrix metalloproteinases (MMPs), particularly matrix metalloproteinase-13 (MMP-13), and ultimately cartilage degradation [4].

In recent years, with the in-depth study of signalling pathways in OA, it has been shown that the expression of proinflammatory factors and matrix-degrading enzymes is upregulated in OA chondrocytes. During the pathological reaction, the MAPK and NF-κB signalling pathways have been shown to play vital roles in the pathogenesis of OA through a series of changes in cartilage damage [5, 6]. MAPK includes extracellular signal regulated kinase (ERK), c-jun terminal kinase (JNK) and the p38 subfamilies, which are involved in regulating various cellular processes, including cell survival, apoptosis, proliferation and the inflammatory response [7]. NF-κB, which is composed of the p65 and p50 subunits, regulates the transcription of many genes and is a key factor in the transcription of many inflammatory genes [8]. Studies have shown that some chemical agents can protect against OA by inhibiting these two pathways [9, 10]. These drugs are superior to the current treatments for OA, such as nonsteroidal anti-inflammatory drugs (NSAIDs) and temporary analgesics, easily resulting in side effects such as gastrointestinal complications [11, 12]. Currently, many antioxidants, especially natural isoflavones, have been reported to have anti-inflammatory and reactive oxygen species (ROS) scavenging functions in many diseases by inhibiting inflammatory signalling pathways [13, 14].

**Fig. 1:**
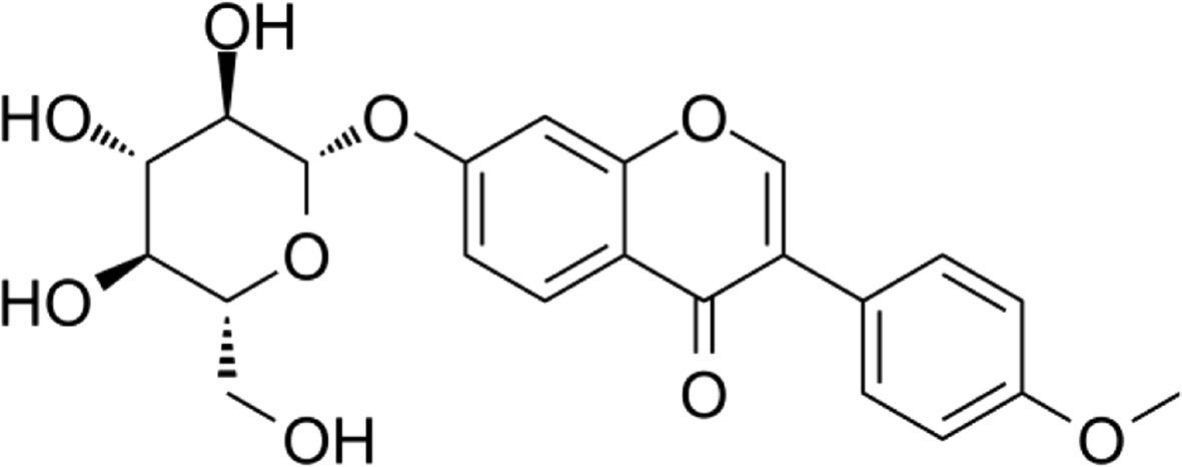
Structure of Ononin

Ononin (Fig. 1) is a representative isoflavone component in traditional Chinese medicines, such as *Astragalus membranaceus*, *Glycyrrhiza uralensis*, *Hedysarum* and *Pueraria lobata*. Ononin has a wide range of biological activities, including antioxidant and anti-inflammatory activities, and can regulate cell proliferation and apoptosis [15, 16]. Ononin has a neuroprotective effect on acute cerebral ischaemia through inhibiting autophagy, apoptosis, and inflammation [17]. Meng et al. [18] confirmed that ononin could alleviate damage in rheumatoid arthritis (RA) by curbing the production of proinflammatory cytokines and inhibiting the NF-κB and MAPK inflammation pathways. These studies showed that ononin has therapeutic effects on inflammatory diseases. However, few studies have ascertained the role of ononin in OA.

In this study, we found that ononin could promote cell proliferation and reduce TNF-α and IL-6 production in an IL-1β-induced OA model in primary chondrocytes. ECM degradation was reversed by inhibiting the expression of MMP-13 and increasing the expression of Collagen II, further showing that ononin could hamper abnormal activation of the MAPK and NF-κB pathways. In conclusion, ononin may be an effective drug for treating OA.

## Materials and methods

### Reagents

Ononin was purchased from MedChemExpress (MCE) (New Jersey, USA). Dimethyl sulfoxide (DMSO), trypsin-EDTA, collagenase II and recombinant rat IL-1β were purchased from Sigma–Aldrich (St. Louis, Missouri, USA). DMSO was used to dissolve ononin, and the final concentration of DMSO in the medium was less than or equal to 1% (which was considered to cause no damage to cells). TNF-α and IL-6 ELISA kits were obtained from R&D Systems (Minneapolis, Minnesota, USA). Foetal bovine serum (FBS) and high glucose Dulbecco’s modified Eagle’s medium (high glucose DMEM) were obtained from HyClone (Grand Island, New York, USA). Rat cartilage-specific antibodies, such as MMP-13 and Collagen II, and the internal reference antibody GAPDH were purchased from Abcam (Cambridge, UK). Antibodies against MAPK and NF-κB pathway-related proteins, such as p-p65/p65, p-IκBα/IκBα, p-ERK/ERK, p-p38/p38 and p-JNK/JNK, and protease and phosphatase inhibitors, were all provided by Cell Signalling Technology (Beverly, Massachusetts, USA). Secondary antibodies, bovine serum albumin (BSA) and 1% penicillin–streptomycin were purchased from Boster (Wuhan, China).

### Cell culture

Five-day-old male Sprague–Dawley (SD) suckling rats were provided by the Animal Laboratory Centre of Guangxi Medical University, Guangxi Zhuang Autonomous Region, China. Chondrocytes were isolated according to the method in a previous study [19]. In brief, cartilage tissues were cut into small pieces of approximately 1 mm^2^ on a superclean workbench, washed with phosphate buffered saline (PBS) several times, incubated with 0.25% trypsin-EDTA in a 37 °C incubator for 0.5 h, and treated with type II collagenase to digest them for another 8 h. Furthermore, the digested cell suspension was transferred to an EP tube and centrifuged at 1000 r/min for 5 min. The supernatant was removed and discarded, and chondrocytes were washed with high glucose DMEM containing 10% FBS 3 times. Finally, the chondrocytes were inoculated in a 25 cm^2^ culture flask containing high glucose DMEM (containing 10% FBS and 1% penicillin–streptomycin) and cultured in a 37 °C incubator with 5% CO_2_. Third-generation chondrocytes in the logarithmic growth stage were used for subsequent experiments.

### Cell viability

We evaluated the effect of ononin on chondrocyte activity by a cell counting kit-8 (CCK-8 kit) assay from Dojindo (Kyushu Island, Japan) according to the experimental methods in a previous study [20]. In brief, third-generation chondrocytes with good growth conditions were selected and inoculated in a 96-well plate at a density of 5 × 10^3^/well. After 1 d of culture, medium containing different concentrations of ononin (0 ~ 100 μM) and with or without IL-1β (10 ng/mL) was added to the cells. After 1 d of culture, 10 μL of CCK-8 reagent was added to each well, and the culture was terminated after being incubated at 37 °C with 5% CO_2_ for 1.5 h. Finally, the absorbance of each well at 450 nm was measured by enzyme-linked immunosorbent assays from Bio–Rad (Richmond, California, USA), and the absorbance value was used to indicate the activity of chondrocytes. Appropriate concentrations of ononin were selected as low, medium and high doses for further study.

### Western blot analysis

Western blotting was used to examine related proteins in chondrocytes, as previously described [20]. In brief, after the experiment was completed, chondrocytes were washed twice with PBS and collected. Then, radioimmunoprecipitation assay (RIPA) lysis solution containing protease and phosphatase inhibitors was added to the chondrocytes and incubated on ice for 30 min. Next, 20 μg protein samples were used for electrophoresis. After that, the proteins in the SDS-polyacrylamide gels were transferred to the activated PVDF membrane by methanol. The edges of the membrane were cut to conform to the size of the antibody incubation boxes, and the membranes were then blocked with 5% BSA for 1 h and incubated with the corresponding diluted primary antibody overnight at 4 °C. After 5 washes with Tris-buffered saline with 0.1% Tween-20 (TBST), the diluted secondary antibody was added and incubated at room temperature for 1 h. After 5 washes with TBST, the enhanced chemiluminescence luminescence (ECL) solution was added. GAPDH was used as the internal reference, and the optical density of the target band was analysed by ImageJ software.

### Immunofluorescence staining

Collagen II and MMP-13 protein levels in chondrocytes were examined by immunofluorescence. The detection method was described in a previous study [20]. In brief, after the cell intervention experiment, the culture medium was discarded, the cells were washed with PBS at room temperature 3 times, and then 4% paraformaldehyde was added and incubated at room temperature for 15 min. After that, the cells were washed 3 times with PBS and incubated in 0.2% Triton X-100 in PBS for 5 min. Then, the cells were blocked with 5% BSA for 30 min. The cells were incubated with Collagen II- or MMP-13-specific antibodies at 4 °C overnight. The cells were washed again 3 times with PBS, and the secondary antibody bound to Cy3 was added dropwise and incubated at 37 °C for 1 h in the dark. Then, the nuclei were stained with DAPI for 10 min. Finally, the protein expression of Collagen II and MMP-13 was observed and photographed by fluorescence microscopy (Evos Flauto, Life Technologies, USA) after the samples were sealed with a fluorescent sealing agent.

### Enzyme-linked immunosorbent assay (ELISA)

The cell culture supernatant and chondrocytes in each group were collected together, and the expression levels of IL-1β and IL-6 were tested according to the instructions of the ELISA kits. In brief, the samples were incubated at 37 °C for 45 min after being added to the reaction well, the washing solution was used to wash each well, and the biotin-labelled antibody was added and reacted for 30 min at 37 °C. After that, streptavidin horseradish peroxidase (HRP) was added and mixed well at 37 °C for 30 min, and then the chromogenic agent was added to avoid discolouration for 15 min. Finally, the termination solution was added to terminate the reaction, and the absorbance of each well at 450 nm was measured by enzyme-linked immunosorbent assays.

### Statistical analysis

GraphPad Prism 9 software was used for statistical analysis and mapping. The measurement data are expressed as the mean ± Sprague-Dawley (SD). Comparisons between multiple groups were performed by one-way analysis of variance (ANOVA), and pairwise comparison was performed with Tukey’s test. *P* < 0.05 indicates that the difference is statistically significant.

## Results

### Effects of Ononin on cell viability

**Fig. 2:**
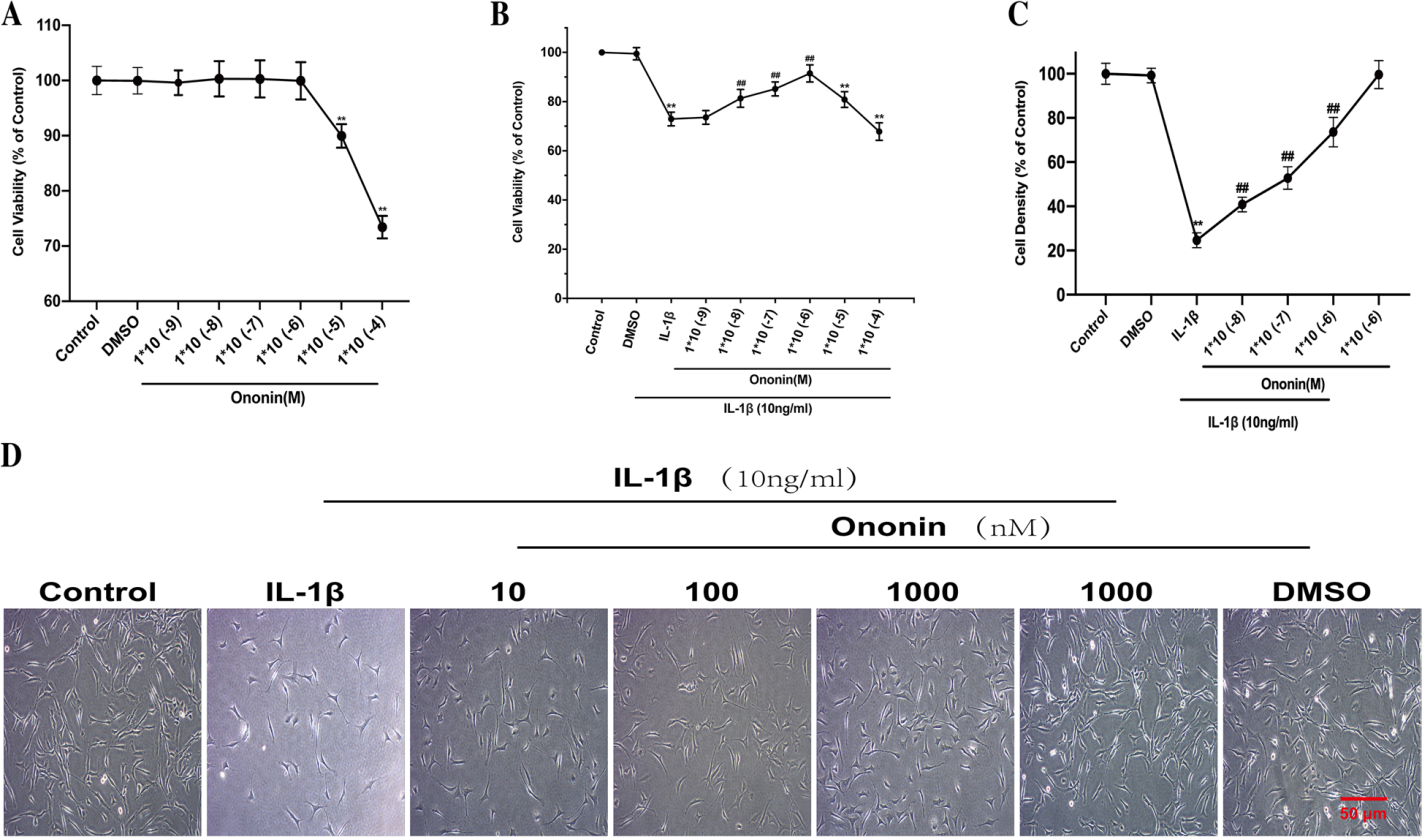
Effects of Ononin on cell viability. (**A**) Rat chondrocytes were intervened on Ononin (1 nM to 100 μM) only or (**B**) with IL-1β (10 ng/ml) for 24 h and cell viability was detected by CCK-8 kit. (**C**) The cell density (% of Control) of different groups was calculated by Image-J software. (**D**) Chondrocytes were treated with Ononin with or without IL-1β (10 ng/ml) and photographed by phase-contrast microscopy (20×) (scale bar: 50 μm). ^**^P < 0.01 compared with control group; ^##^*P* < 0.01 compared with IL-1β group

The toxicity of ononin on chondrocytes was assessed by a CCK-8 kit. Cells were cultured with ononin at concentrations ranging from 1 nM to 100 μM with or without IL-1β (10 ng/ml) for 24 h. As shown in Fig. 2A, compared with the control group, the DMSO group (≤ 1‰) exhibited no significant toxicity, as measured by cell proliferation (*P* > 0.05). Conversely, the activity of IL-1β-induced chondrocytes was decreased (*P* < 0.01), as shown in Fig. 2B. Ononin at concentrations of 10, 100, and 1000 nM for 24 h was not cytotoxic to chondrocytes in the presence or absence of IL-1β (10 ng/ml). However, cell viability was inhibited by high concentrations of ononin (≥ 10 μM) (*P* < 0.01). Interestingly, in IL-1β-induced chondrocytes treated with low doses of ononin (10 to 1000 nM), cell viability gradually increased compared with that in the control group (P < 0.01). Finally, we used phase-contrast microscopy and observed that the cells in the control group and the ononin alone group were highly dense, while the cell density in the IL-1β group was comparatively sparse, but the cell density gradually increased in response to ononin and recovered to approximately 75% in the 1000 nM ononin group compared with control group, suggesting that it has a strong promoting effect on cell activity, Fig. 2C, D. Therefore, ononin at 10, 100 and 1000 nM were selected for subsequent experiments.

### Effects of Ononin on IL-1β-induced production of the inflammatory cytokines TNF-α and IL-6 in rat chondrocytes

**Fig. 3:**
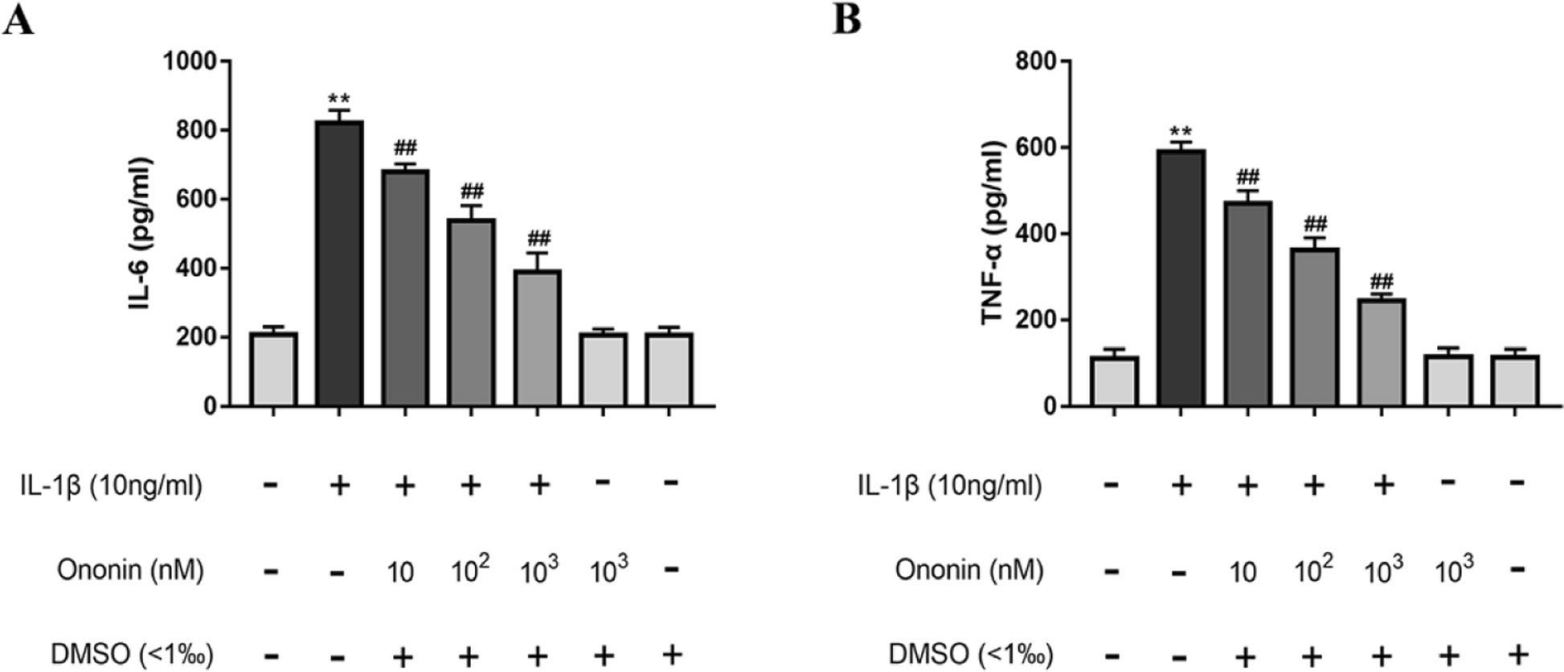
Effects of Ononin on IL-1β-induced production of TNF-α and IL-6 in rat chondrocytes. The cell culture supernatant and chondrocytes of each group were collected together. (**A**, **B**) expression levels of TNF-α and IL-6 levels were accessed by ELISA kits. All experiments were performed in 3 times and data were expressed by mean ± SD. ^**^P < 0.01 compared with control group; ^##^*P* < 0.01 compared with IL-1β group

Next, we studied the effect of ononin on the expression of certain key cytokines in the pathogenesis of OA. To examine whether ononin could inhibit the upregulation of TNF-α and IL-6 induced by IL-1β, ELISA kits were used to evaluate the levels of these inflammatory factors. As shown in Fig. 3, IL-1β significantly induced the release of the inflammatory factors TNF-α and IL-6 by chondrocytes. In addition, 10, 100 and 1000 nM ononin markedly suppressed the production of TNF-α and IL-6 compared to that in the IL-1β group. In particular, the levels of TNF-α and IL-6 in the 1000 nM ononin group were reduced 2-fold and 3-fold, respectively, compared with those in the control group; moreover, this downregulation showed a decreasing trend with increasing ononin concentrations. In brief, these results suggested that ononin reduced the release of the inflammatory cytokines TNF-α and IL-6 in IL-1β-induced rat chondrocytes.

### Effects of Ononin on IL-1β-induced cartilage degradation and overexpression of MMP-13 in chondrocytes

**Fig. 4:**
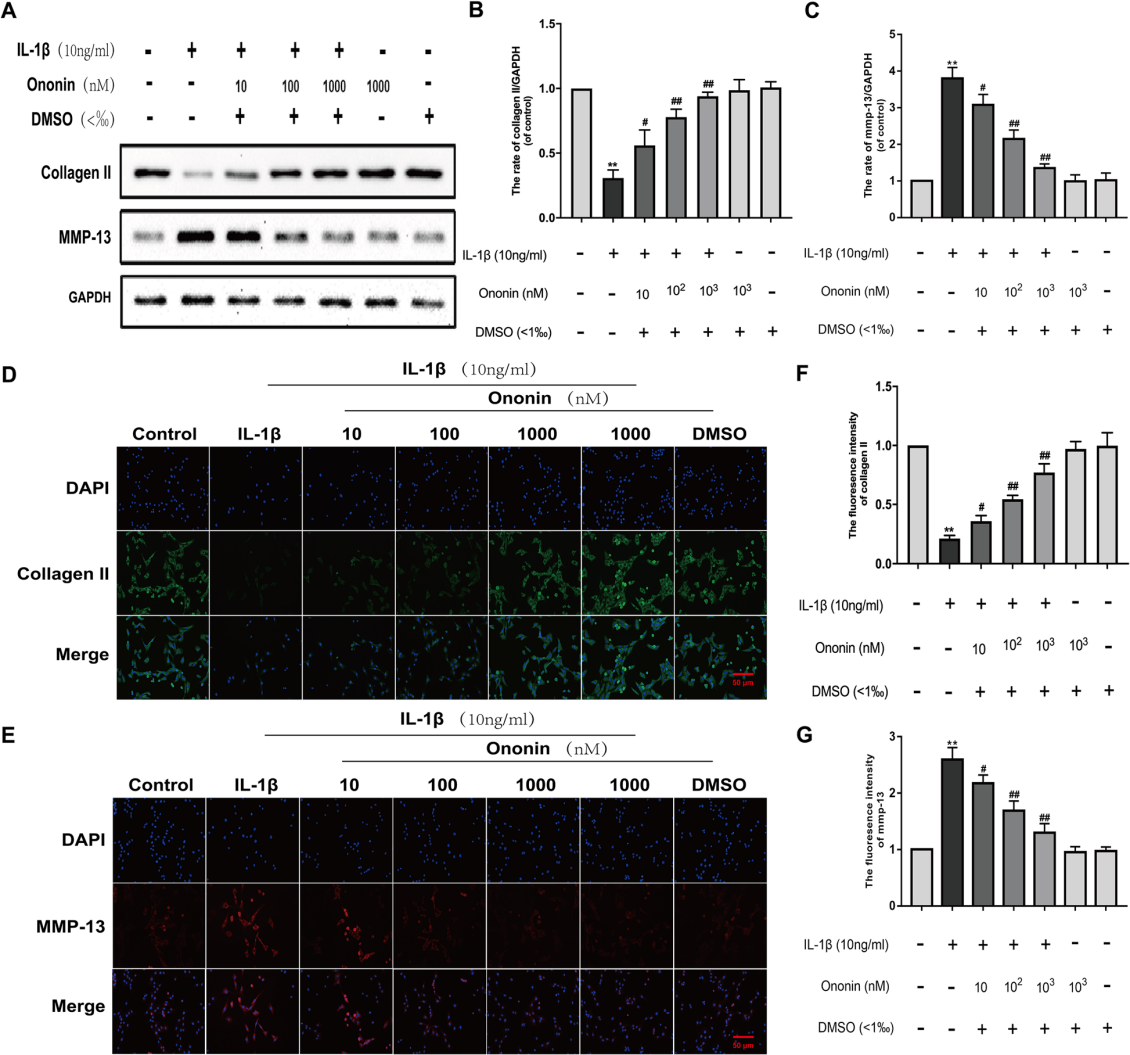
Effects of Ononin on IL-1β-induced ECM degradation in Rat Chondrocytes. (**A**) Expression levels of Collagen II and MMP-13 were detected by Western Blot. The samples derive from the same experiment and that different gels were processed in parallel. (**B**, **C**) The expression level of target proteins was expressed by the ratio of grey value of target band to GAPDH band by Image-J software. (**D**) Immunofluorescence staining of Collagen II protein (green) and nucleus (blue) was labelled with DAPI and photographed by fluorescence microscope (20×) (scale bar: 50 μm). (**E**) Immunofluorescence staining of MMP-13 protein (red) and nucleus (blue) was labelled with DAPI and photographed by fluorescence microscope (20×) (scale bar: 50 μm). (**F**, **G**) The summary data of fluorescence intensity of Collagen II and MMP-13 in situ. All experiments were performed in 3 times and data were expressed by mean ± SD. ^*^P < 0.05 compared with control group; ^#^*P* < 0.05 compared with IL-1β group; ^**^P < 0.01 compared with control group; ^##^*P* < 0.01 compared with IL-1β group

Collagen II is commonly used to assess cartilage tissue metabolism, and MMP-13 is the major cartilage degradation enzyme associated with the progression of OA [20]. The protein expression levels of Collagen II and MMP-13 were evaluated by Western blotting (Fig. 4A) and immunofluorescence staining (Fig. 4D, E). In our study, chondrocytes were treated with ononin with or without IL-1β for 24 h. As shown in Fig. 4B-C and F-G, IL-1β noticeably inhibited the expression of Collagen II and upregulated the expression of MMP-13. However, 10, 100 and 1000 nM ononin effectively reversed the degradation of Collagen II and the overexpression of MMP-13. Furthermore, ononin ameliorated IL-1β-induced adverse effects on Collagen II and MMP-13 protein expression in a concentration-dependent manner. The immunofluorescence staining results were consistent with the Western blot results, in the same light, in the 1000 nM ononin group, ononin decreased the expression of MMP-13 by approximately 2.9 times and Collagen II was restored to 90% compared with those in the IL-1β group, showing the ability to repair cartilage damage.

### Effects of Ononin on IL-1β-induced MAPK and NF-κB Signalling activation in rat chondrocytes

**Fig. 5:**
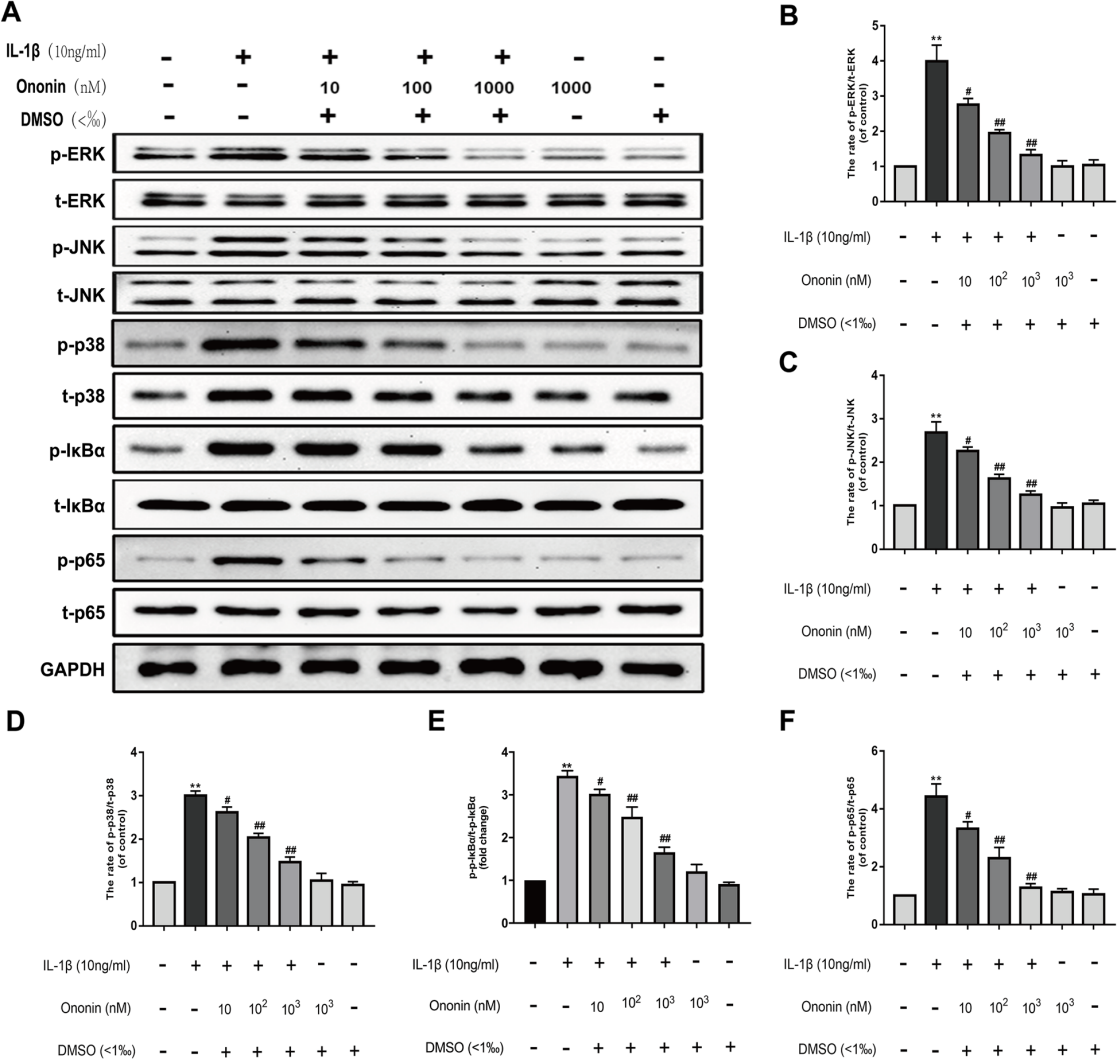
Effects of Ononin on MAPK and NF-κB signalling pathways. (**A**) Proteins of p-ERK/ERK, p-JNK/JNK, p-p38/ p38, p-IκBα/IκBα, and p-p65/p65 were detected by Western Blot. The samples derive from the same experiment and that different gels were processed in parallel. (**B**-**F**) The expression level of target proteins was expressed by the ratio of grey value of target band to the total of ERK, p38, JNK, IκBα and p65 were used as the internal control respectively by Image-J software. All experiments were performed in 3 times and data were expressed by mean ± SD. ^*^P < 0.05 compared with control group; ^#^*P* < 0.05 compared with IL-1β group; ^**^P < 0.01 compared with control group; ^##^*P* < 0.01 compared with IL-1β group

The MAPK and NF-κB signalling pathways play key roles in the occurrence and development of OA [21, 22]. To explore the effect of ononin on IL-1β-induced activation of the MAPK and NF-κB pathways in rat chondrocytes, first, the levels of phosphorylated ERK, JNK and p38 and their corresponding total protein levels were measured by Western blotting (Fig. 5A). As shown in Fig. 5B-D, the ratios of p-ERK/ERK, p-JNK/JNK and p-p38/p38 in the IL-1β group were significantly higher than those in the control group (*P* < 0.01). In contrast, ononin at concentrations of 10, 100 and 1000 nM effectively inhibited the phosphorylation of ERK, JNK and p-p38 (*P* < 0.05). In the 1000 nM ononin group, the ratios of p-ERK/ERK, p-JNK/JNK and p-p38/p38 decreased by 3.9 times, 2.1 times and 2 times, respectively, compared with those in the IL-1β group.

Then, we used the same method to examine the related proteins in the NF-κB pathway, including p-IκBα/IκBα and p-p65/p65 (Fig. 5A). As shown in Fig. 5E-F, ononin at concentrations of 10, 100 and 1000 nM could also inhibit the IL-1β-induced upregulation of the ratios of p-IκBα/IκBα and p-p65/p65, which decreased 2.5 times and 3.5 times, respectively, with high concentrations of ononin (1000 nM ononin group). In summary, these two activated pathways can be partially restored by ononin in a dose-dependent manner.

## Discussion

OA is a degenerative joint disease. The core pathological change in OA is the inflammatory reaction in the joints, which further leads to the loss of articular cartilage to different degrees [23, 24]. Therefore, inflammation is an important link in the pathogenesis of OA. There is still no effective treatment to prevent the progression of OA. Therefore, it is urgent to develop more effective OA drugs. Ononin, a natural isoflavone, is widely distributed in *Astragalus membranaceus, Glycyrrhiza uralensis, Hedysarum and Pueraria lobata* [25, 26]. Based on previous studies, ononin has anti-inflammatory and apoptotic effects [18, 27, 28]. In this study, we used IL-1β (10 ng/ml)-induced chondrocytes as an OA model in vitro and hypothesized that ononin mediated inflammation during OA through targets or signalling pathways. Here, we showed that ononin could inhibit the IL-1β-induced expression of the cytokines TNF-α and IL-6. Furthermore, ononin also alleviated the IL-1β-induced expression of MMP-13 and restored the degradation of collagen II in rat chondrocytes. Finally, ononin could play a protective role in chondrocytes by regulating the MAPK and NF-κB pathways. Overall, our results suggest that ononin may be a promising therapeutic agent for OA.

Many studies have elucidated that IL-1β, IL-6, TNF-α and IL-17 are the key proinflammatory cytokines leading to the pathogenesis of OA and are found at high levels in OA chondrocytes [29, 30]. Therefore, regulating the receptors of these cytokines may be favourable for the treatment of OA. Recent studies have widely reported that curcumin and resveratrol are potential therapeutic drugs for OA that inhibit the inflammatory response and oxidative stress in chondrocytes [31, 32]. Studies have also shown that ononin can protect LPS-induced RF inflammatory cells and RAW 264.7 cells from damage [18, 27]. In our study, ononin significantly inhibited the IL-1β-mediated enhancement of the inflammatory factors TNF-α and IL-6 in rat chondrocytes. Furthermore, various studies have confirmed that there is a high level of MMP-13 in damaged articular cartilage. Generally, MMP-13 is the main enzyme that targets cartilage catabolism and plays a crucial role in the process of articular cartilage degradation [33]. In general, proinflammatory factors such as TNF-α and IL-6 stimulate the production of MMPs and inhibit the synthesis of ECM [34–36]. In our study, we revealed that IL-1β-induced chondrocytes had increased expression of MMP-13 and inhibited expression of Collagen II, further demonstrating that ononin significantly ameliorated damage in a dose-dependent manner. Previous studies have shown that a growing number of Chinese herbal compounds also have anti-inflammatory effects against OA, which was consistent with our results. For instance, betulin can inhibit the degradation of extracellular matrix by downregulating the expression of MMP-13 and upregulating the expression of collagen II [37]. Another agent, xanthohumol, a natural prenylflavonoid with anti-inflammatory and antioxidant activities, has also been shown to effectively protect against ECM degradation by ameliorating MMP-13 expression in OA chondrocytes [38]. In conclusion, our data suggested that ononin could restore the imbalance in cartilage matrix anabolism.

In recent years, with the in-depth study of signalling pathways in the development of OA, the MAPK and NF-κB pathways have been increasingly shown to be important regulators of the inflammatory response, catabolism and ECM degradation during the OA process [39, 40]. MAPK is a serine/threonine protein kinase. Under inflammatory conditions, ERK, JNK and p38 are activated, and their phosphorylation levels are upregulated, thus stimulating downstream transcription factors and leading to the production of proinflammatory cytokines and the inflammatory response [41]. Akhtar et al. [42] confirmed that inflammatory factors such as IL-1β can induce the overexpression of miR-27b and promote the upregulation of MMP-13 by inducing JNK phosphorylation in melanoma. Sonder et al. [43] found that p38 MAPK, p44/42 and src tyrosine kinase inhibitors can inhibit the degradation of ECM by inhibiting MMPs, although p44/42 inhibitors are essential for delaying the degradation of proteoglycan. In addition, aucubin is a natural compound derived from plants that can inhibit IL-1β-mediated p65 phosphorylation and nuclear translocation, suggesting that aucubin may have anti-inflammatory and cartilage protective properties through the NF-κB signalling pathway [44]. Consistent with these studies, the present study showed that chondrocytes stimulated with IL-1β for 24 h had significantly increased phosphorylation of ERK, JNK and p38. However, when ononin was added to IL-1β-induced chondrocytes, the phosphorylation of these proteins was inhibited in a dose-dependent manner. This finding suggests that ononin may regulate the inflammatory response and ECM degradation by inhibiting the IL-1β-activated MAPK pathway.

The NF-κB signalling pathway is present in all kinds of cells, mainly in the form of an inactive p65 subunit. The upstream signal is transmitted to IκB kinase. Then, IκB kinase phosphorylates I-κB and exposes nuclear factor κB (p65), which is inhibited by I-κB, after which p65 is activated and phosphorylated. Finally, the phosphorylated protein is dissociated, and p65 is translocated into the nucleus, thus activating the inflammatory response and leading to the secretion of a series of proinflammatory cytokines [45]. Moreover, activation of the NF-κB pathway is necessary for chondrocytes to express MMPs, trigger the inflammatory response and exacerbate cartilage destruction [46]. Recently, resveratrol, a flavonoid with a stilbene structure, has been shown to block the NF-κB pathway to mitigate chondrocyte inflammation and ameliorate IL-1β-induced chondrocyte injury [32, 47]. In addition, the aqueous extract of *Anthriscus sylvestris* leaves, an agent similar to that in our study, can protect cartilage against OA by inhibiting IL-1β-induced MAPK phosphorylation and NF-κB p65 subunit translocation to the nucleus [48]. Moreover, Dai et al. [49] claimed that after anthocyanin treatment, the NF-κB signalling pathway was significantly inhibited, resulting in reductions in IL-1β and TNF-α and the expression of matrix metalloproteinase, which was shown to significantly alleviate the occurrence of pain and inflammation induced by OA. In our study, compared with that in the control group, the expression of p-IκBα in IL-1β-activated chondrocytes was increased, which led to the release of p65. Then, p65 translocated into the nucleus and activated inflammation, which upregulated the expression of secreted inflammatory factors and ECM decomposition. However, these results were significantly reversed in a dose-dependent manner after treatment with ononin.

## Conclusion

The findings in this study were the first to reveal that ononin had clear anti-inflammatory therapeutic effects on OA. As we have discovered, inhibiting the inflammatory response and reducing ECM degradation may be the basis of the protective mechanism of ononin, which may further occur via the regulation of the MAPK and NF-κB signalling pathways. These results suggest that ononin could become a promising treatment against OA.

## Supplementary Information

**Additional file 1.**

## Data Availability

All data generated or analyzed during this study are included in this published article and its important information files.
