# Supplementary material for: Ononin ameliorates inflammation and cartilage degradation in rat chondrocytes with IL-1β-induced osteoarthritis by downregulating the MAPK and NF-κB pathways
Source: BMC Complement Med Ther. 2022 Jan 27;22:25. doi: 10.1186/s12906-022-03504-5 (PMC8793192; doi:10.1186/s12906-022-03504-5)
Supplement: Supplementary file 1 — Additional file 1. [file 12906_2022_3504_MOESM1_ESM.pdf]

Ononin Ameliorates Inflammation and Cartilage Degradation in Rat Chondrocytes with IL-1β-induced Osteoarthritis by downregulating the MAPK and NF-κB Pathways

Fang Xu[1,2], Liang-Jun Zhao[3], Ting Liao[4], Zhao-Cong Li[2,5], Lei-Lei Wang[2,5], Pan-Yu Lin[1], Rui Jiang[1], Qing-Jun Wei[1*]

* Correspondence: weiqingjungxnn@163.com

[1]Department of Orthopaedics Trauma and Hand Surgery, The First Affiliated Hospital of Guangxi Medical University, Nanning, 530021, China.

[2]Guangxi Colleges and Universities Key Laboratory of Prevention and Control of Highly Prevalent Diseases, Guangxi Medical University, Nanning, 530021, China.

[3]Department of Bone and Joint Surgery, The First Affiliated Hospital of Guangxi Medical University, Nanning, 530021, China.

[4]Department of Endocrinology, Liuzhou Municipal Liutie Central Hospital, Liuzhou, 545007, China.

[5]Department of Toxicology, School of Public Health, Guangxi Medical University, Nanning, 530021, Guangxi, China.

Fang Xu and Liang-Jun Zhao contributed equally to this work.

Full list of author information is available at the end of the article.

Supplementary Information

the full length original blot

Collegan II:

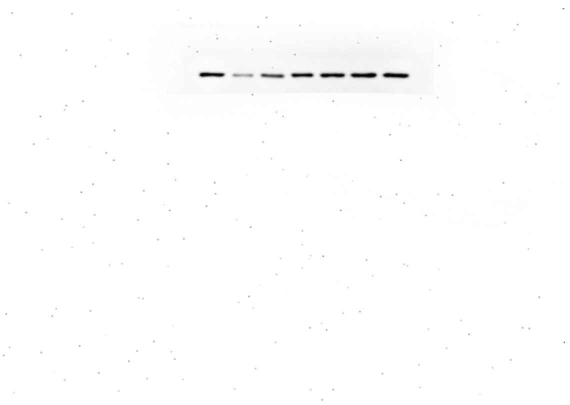

Figure 1 a. the original blot of collegan II.

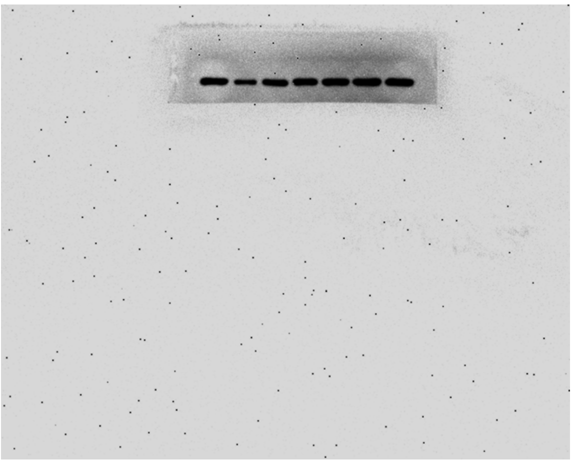

Figure 1 b. the original blot of collegan II (another exposure image).

MMP-13:

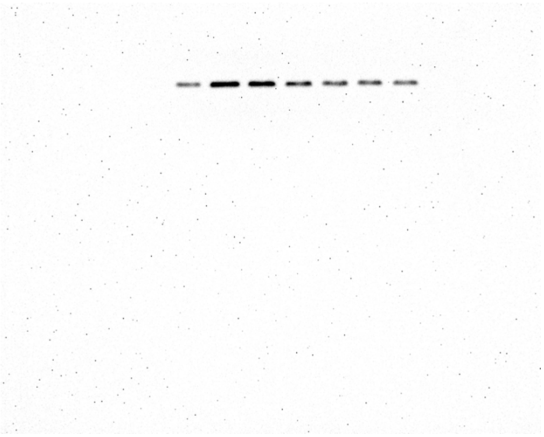

Figure 2 a. the original blot of MMP-13.

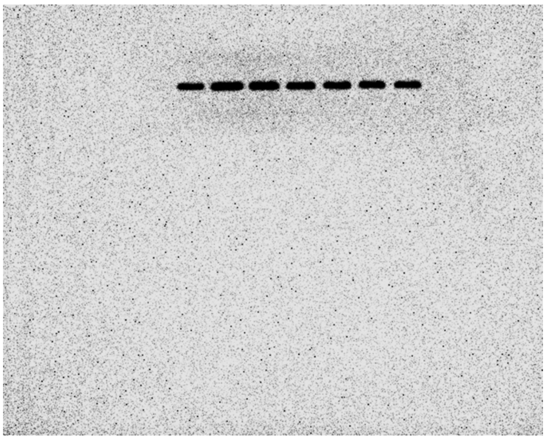

Figure 2 b. the original blot of MMP-13 (another exposure image).

GAPDH (for collegan II and MMP-13 proteins):

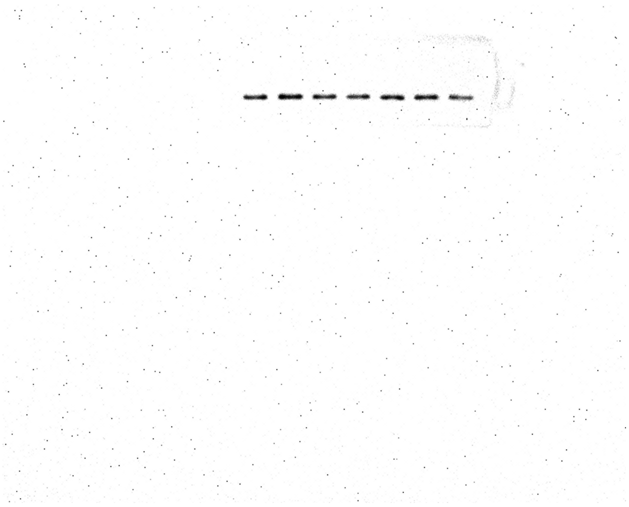

Figure 3 a. the original blot of GAPDH (for collegan II and MMP-13 proteins).

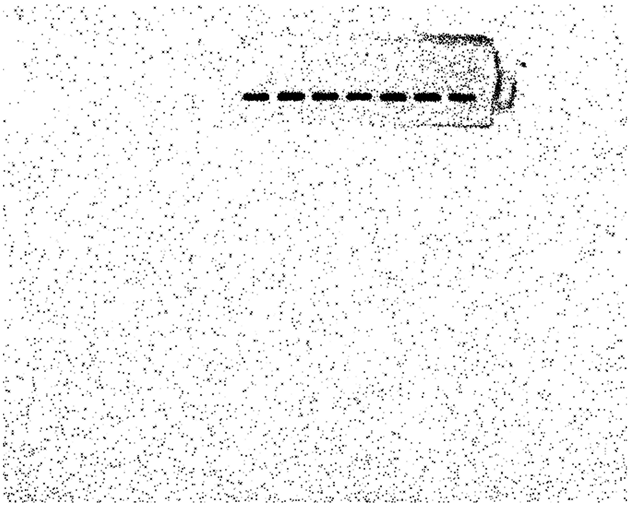

Figure 3 b. the original blot of GAPDH (for collegan II and MMP-13 proteins) (another exposure image).

p-ERK:

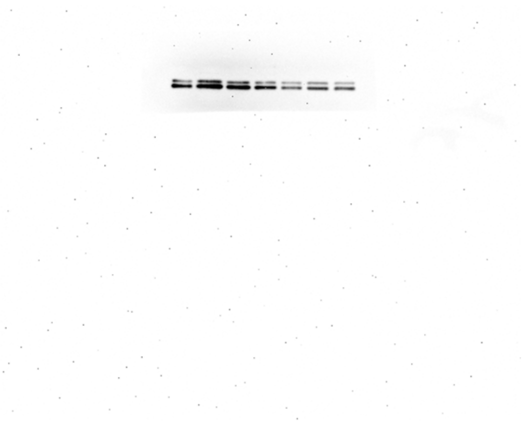

Figure 4 a. the original blot of p-ERK.

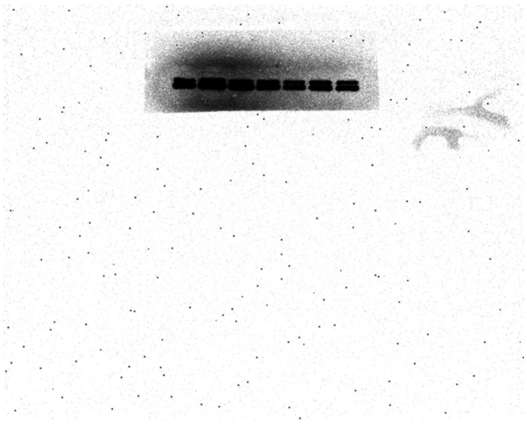

Figure 4 b. the original blot of p-ERK (another exposure image).

t-ERK:

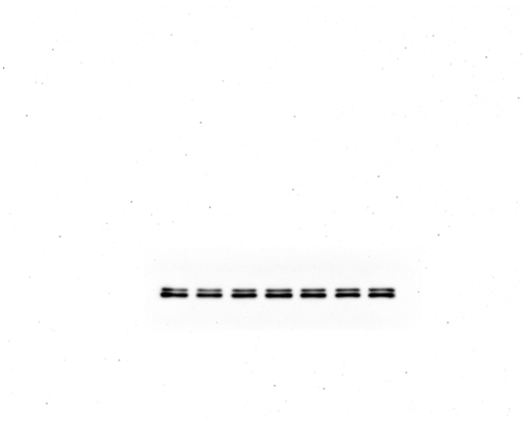

Figure 5 a. the original blot of t-ERK.

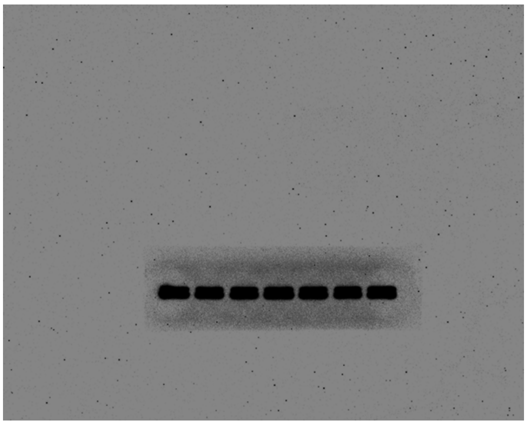

Figure 5 b. the original blot of t-ERK (another exposure image).

p-JNK:

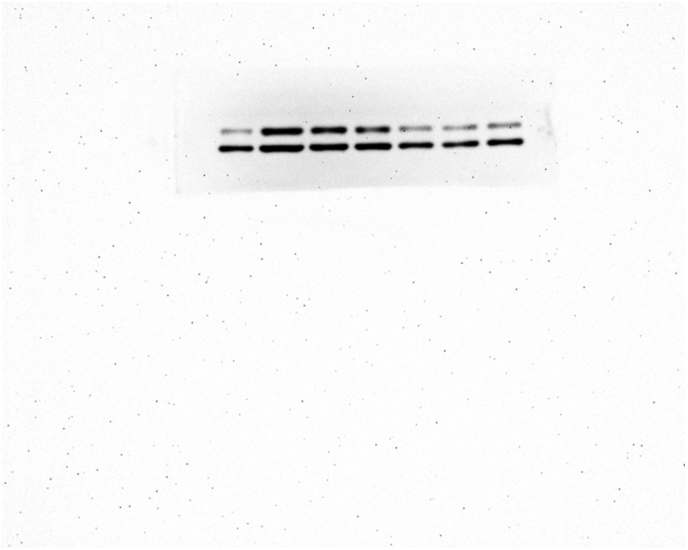

Figure 6. the original blot of p-JNK.

t-JNK:

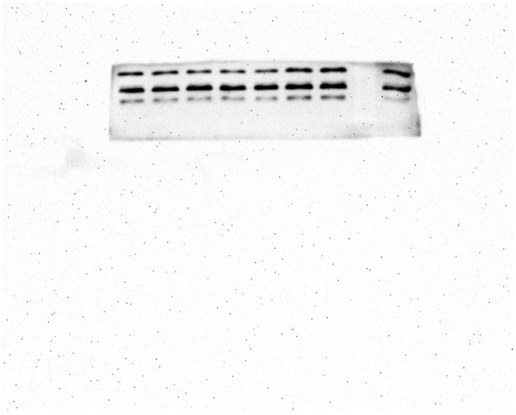

Figure 7. the original blot of t-JNK.

p-P38 (bottommost blot):

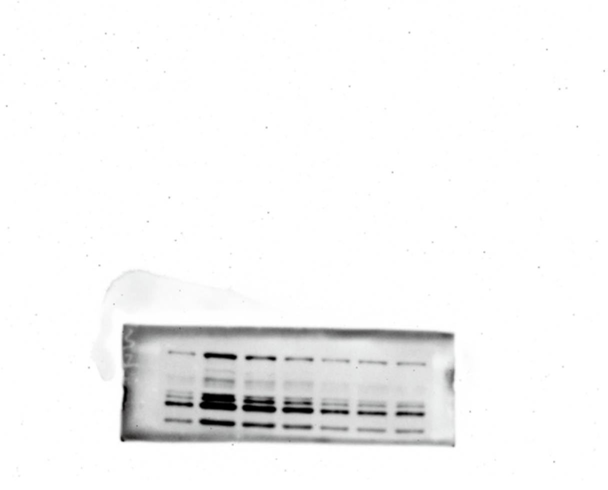

Figure 8. the original blot of p-P38 (bottommost of the blot).

t-P38:

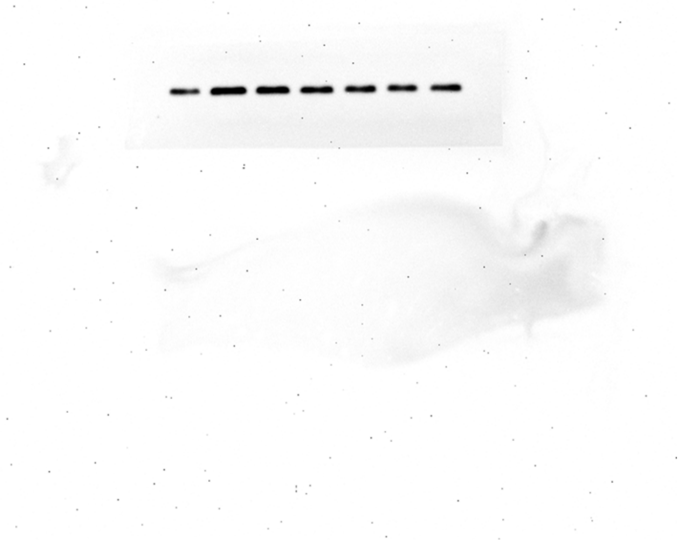

Figure 9 a. the original blot of t-P38.

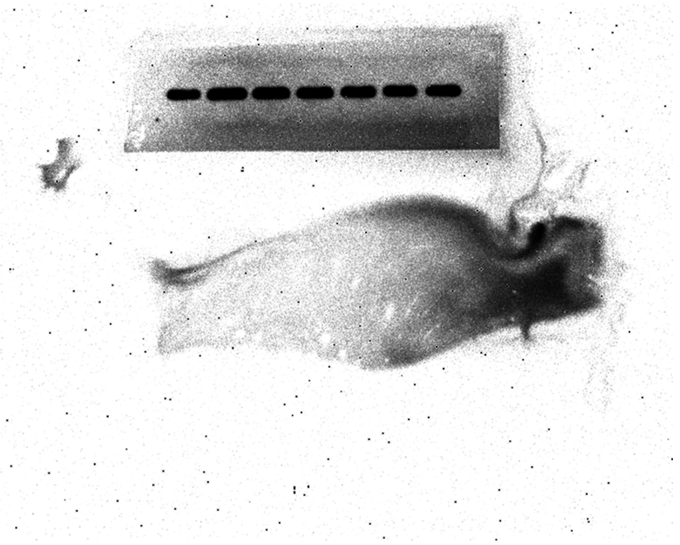

Figure 9 b. the original blot of t-P38 (another exposure image).

p-IϰBα:

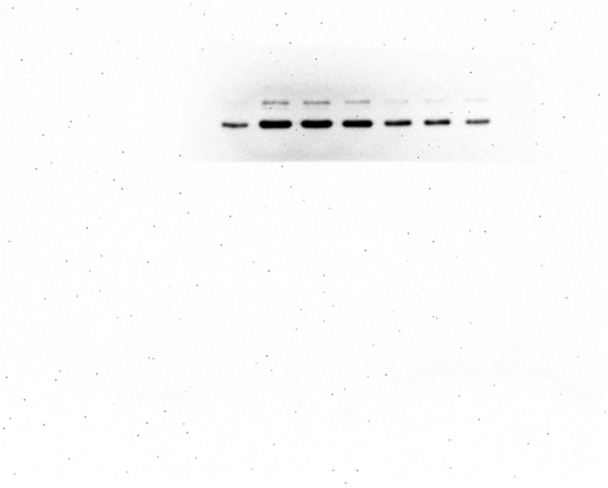

Figure 10. the original blot of p-IϰBα.

t-IϰBα:

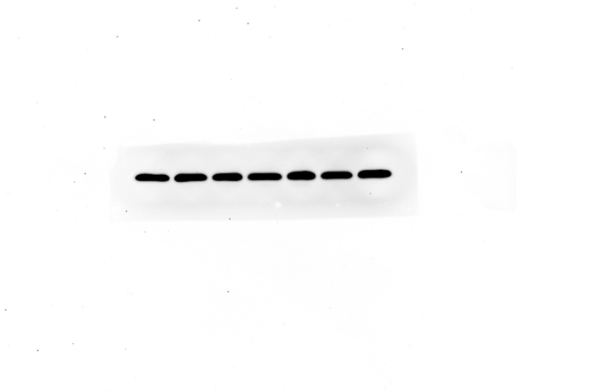

Figure 11 a. the original blot of t-IϰBα.

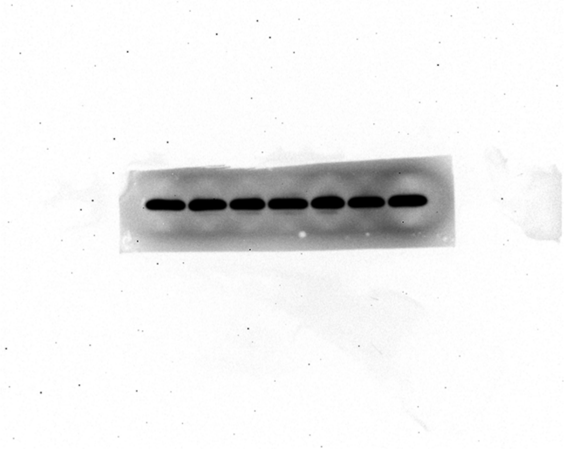

Figure 11 b. the original blot of t-IϰBα (another exposure image).

p-P65 (upmost blot):

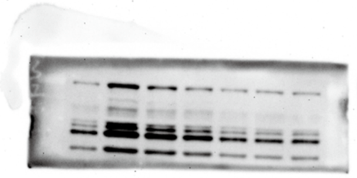

Figure 12. the original blot of p-P65 (upmost blot).

t-P65:

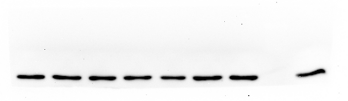

Figure 13 a. the original blot of t-P65.

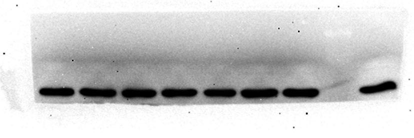

Figure 13 b. the original blot of t-P65 (another exposure image).

GAPDH (for p-ERK/t-ERK, p-JNK/t-JNK, p-P38/t-P38, p-IκBα/t-IκBα and p-P65/t-P65 proteins):

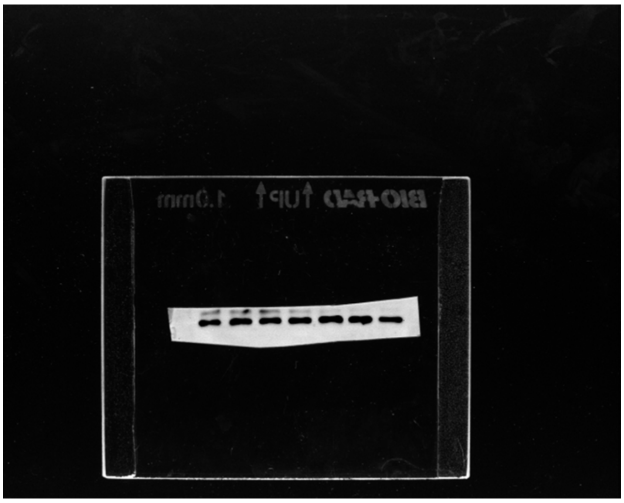

Figure 14. the original blot of GAPDH (for p-ERK/t-ERK, p-JNK/t-JNK, p-P38/t-P38, p-IκBα/t-IκBα and p-P65/t-P65 proteins).
